# Bortezomib-Induced Bronchiolitis Obliterans Organizing Pneumonia

**DOI:** 10.1155/2012/430141

**Published:** 2012-10-21

**Authors:** E. Vandeix, F. Favard, N. Pichon, M. Delage-Corre, B. Melloni, M. Clavel

**Affiliations:** ^1^Medical-Surgical Intensive Care Unit, Dupuytren University Hospital, 2 Avenue Martin Luther King, 87000 Limoges, France; ^2^Department of Pulmonology, Dupuytren University Hospital, 2 Avenue Martin Luther King, 87000 Limoges, France; ^3^Department of Pathology, Dupuytren University Hospital, 2 Avenue Martin Luther King, 87000 Limoges, France

## Abstract

*Introduction*. Bortezomib is a proteasome inhibitor indicated for the treatment of multiple myeloma patients. The most frequent side effects are gastrointestinal and neurological. Serious pulmonary complications have been described rarely. *Observation*. This case involves a 74-year-old man suffering from IgG Kappa myeloma treated with bortezomib, melphalan, and dexamethasone. After administering chemotherapy, the patient developed an acute respiratory distress syndrome (ARDS). A surgical pulmonary biopsy proved the existence of bronchiolitis obliterans organizing pneumonia (BOOP) lesions. Systemic corticotherapy led to a rapid improvement in the patient's condition. *Conclusion*. This is the first reported histologically confirmed case of bortezomid-induced BOOP. Faced with severe respiratory symptoms in the absence of other etiologies, complications due to bortezomid treatment should be evoked and corticotherapy considered.

## 1. Introduction

Bortezomib is a proteasome inhibitor. In association with melphalan and prednisone, it is indicated for the treatment of nonpreviously treated multiple myeloma patients or for the treatment of relapsed multiple myeloma who are not eligible for a bone marrow transplant. The most frequent side effects are gastrointestinal and neurological. Rare cases of acute pulmonary complications have been reported.

## 2. Observation

**Figure 1:**
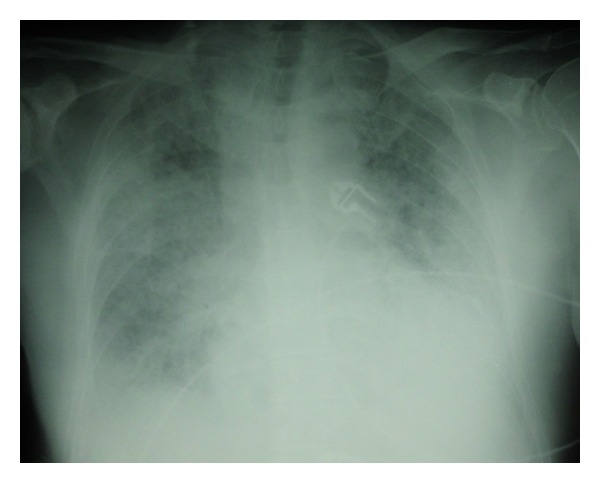
Postero anterior radiograph of the chest obtained on admission.

**Figure 2:**
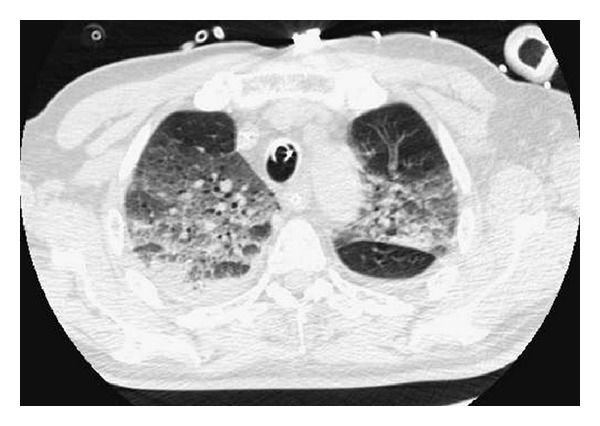
CT scan of the chest obtained on Day 1 showing diffuse bilateral alveolar infiltrate.

A 74-year-old male patient suffering from an IgC Kappa multiple myeloma. The *performans status* of the patient before treatment was 1. Treatment with bortezomib (1.3 mg/m², Day 1, Day 8, Day 15, and Day 22), melphalan (0.15 mg/kg/day for 4 days), and dexamethasone (20 mg/day for 4 days) was instigated. During the treatment period, the patient also received prophylaxis with trimethoprim/sulfamethoxazole and valacyclovir. The patient had no respiratory history and was neither a current or previous smoker. The initial chest X-ray was normal. Three days after his eighth course of chemotherapy, the patient was hospitalised for a cough, a febrile dyspnea associated with a low quantity of hemoptoic expectoration. Auscultation revealed crackles in both pulmonary bases. The patient rapidly developed acute respiratory distress (SaO_2_ 89%) on mask with reservoir bag 15 L/min oxygen. The chest X-ray showed a diffuse bilateral alveolar-interstitial syndrome predominantly in the lower lobes (Figure 1). The PaO_2_/FiO_2_ ratio was 70. The transthoracic echocardiography was normal. A biological inflammatory syndrome was present. Noninvasive ventilation was performed. Faced with a lack of improvement, orotracheal intubation proved necessary to put the patient under mechanical ventilation. A CT scan revealed a bilateral interstitial alveolar infiltration, predominantly to the bases, a low amount of right pleural effusion, lesions of emphysema, and an absence of adenomegaly (Figure 2). Bacteriological blood and urine samples were negative. A bronchoalveolar lavage (BAL) was performed. Intravenous dual antibiotic therapy using 2 g/day of ceftriaxone and sulfamethoxazole (15 mg/kg per day) with trimethoprim (75 mg/kg/day) was started the day of intubation.

**Figure 3:**
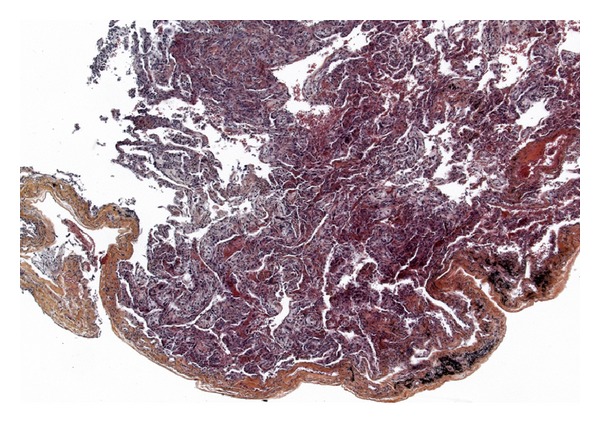
Open lung biopsy performed on day 6 (HES × 50): lesions of bronchiolitis obliterans with organizing fibroblastic polyps in alveoli.

**Figure 4:**
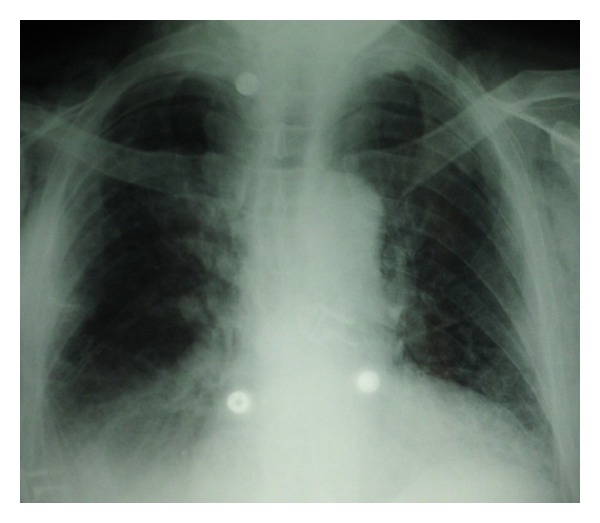
Postero anterior radiograph of the chest on Day 12.

Despite protective ventilation and antibiotic therapy, the clinical development was a persistent and severe ARDS. Due to the negative etiological report and lack of patient improvement, an open lung biopsy was performed on the sixth day of mechanical ventilation. The bacteriological, virological, mycological, and parasitological examination of the lung biopsy was negative. The anatomopathological examination found dentoalveolar vegetating fibromas typical of BOOP (Figure 3). Corticosteroid therapy using intravenously administered methylprednisolone was initiated at a dose of 1 mg/kg/day. Clinical, gasometric, and radiological improvement in few days enabled extubation and mechanical ventilation to be discontinued on the twelfth day (Figure 4). Oxygen therapy was stopped on Day 15.

## 3. Discussion

In the absence of other infectious or drug related etiologies, our observation suggests that bortezomib can cause ARDS secondary to BOOP lesions. Rare clinical cases have already been reported. Miyakoshi et al. [1] have described episodes of acute respiratory distress in four Japanese patients treated with bortezomib. Half of the patients survived. Dun et al. [2] have described fives lethal cases of pulmonary complications associated with bortezomib in China. These are the two series of cases with the largest number of patients. Zappasodi et al. [3] have reported an observation on an Italian patient developing respiratory distress after administration of bortezomib. The CT scan suggested BOOP. Histological confirmation could not be obtained. The patient improved under corticosteroid therapy. Kang et al. [4] have reported the case of a Chinese patient displaying acute dyspnoea and interstitial pneumopathy on the CT scan after administration of bortezomib-thalidomide-dexamethasone. A surgical pulmonary biopsy was performed, the anatomopathological analysis of which revealed a nonspecific diffuse interstitial pneumopathy. This was the first case confirmed by histology after administration of bortezomib-thalidomide. Systemic corticosteroid therapy led to clinical and radiological improvement. In most of the published cases, the respiratory distress occurred after the first or second cycle. The occurrence of severe pulmonary complications after the third bortezomib cycle has been described by Boyer et al. [5]. Another case published by Zhou et al. [6] relates to a patient developing acute respiratory insufficiency several hours after the first injection of bortezomib, rapidly leading to death. Our observation is the first to describe such a late onset of pulmonary symptoms after starting chemotherapy. Amongst these clinical cases, some patients had not received dexamethasone at the time of the bortezomib injection. Gotoh et al. [7] have shown that the risk of pulmonary complications under bortezomib fell when bortezomib was combined with dexamethasone. Phase IV clinical trials on 666 patients who had received bortezomib published by Narimatsu et al. [8] experienced 3.6% of pulmonary complications and a 0.5% death rate attributable to these complications. The pulmonary toxicity mechanism of bortezomib remains unknown. Bortezomib is a selective inhibitor of proteasome 26S and blocks activation of NF-*κ*B (cellular transcription nuclear factor). Several pathogenicity pathways have been suggested [9] in the development of bortezomib-related pulmonary complications.

- Activation of NF-*κ*B-related proinflammatory factors such as IL-6 and TNF-*α*.
- Accumulation of bortezomib and/or its metabolites, altering multiple signalling pathways.

The first hypothesis would explain the rapid response to corticosteroid therapy obtained in some patients. The patient did not receive any other treatment which could lead to pulmonary toxicity. Only a long-term treatment with melphalan has been described to generate pulmonary fibrosis, and our patient received a short treatment with low doses [10, 11]. Despite the precarious respiratory condition, it was decided to perform a surgical pulmonary biopsy to investigate the etiology. This procedure had an impact on our therapeutic care, and no complications were noted. This is a new example of the feasibility and usefulness of open lung biopsy in a patient with ARDS to determine etiology and to govern treatment [12].

## 4. Conclusion

Bortezomib seems to be the source of sometimes severe respiratory complications. This is the first reported histologically confirmed case of BOOP as a side effect of bortezomib administration. This case suggests that vigilance is required in patients treated with bortezomib. In this context, systemic corticotherapy will have to be considered faced with acute respiratory symptoms in the absence of other etiologies.

## Abbreviations

BOOP: Bronchiolitis obliterans organizing pneumonia
ARDS: Acute respiratory distress syndrome
BAL: Bronchoalveolar lavage.
